# Case Report: Diffuse Large B-Cell Lymphoma in the Wall of a Thymic Cyst

**DOI:** 10.3389/fsurg.2022.788047

**Published:** 2022-02-02

**Authors:** Apostolos C. Agrafiotis, Ioannis Paralikas, Kanellos Giakoumakis, Sotirios D. Moraitis

**Affiliations:** ^1^Department of Thoracic Surgery, Saint-Pierre University Hospital, Université Libre de Bruxelles (ULB), Brussels, Belgium; ^2^Departmentof Thoracic Surgery, Athens Naval and Veterans Hospital, Athens, Greece; ^3^Department of Anesthesiology, Athens Naval and Veterans Hospital, Athens, Greece; ^4^Joint Corps Armed Forces Cardiac Surgery Department, 401 Hellenic Army Hospital, Athens, Greece

**Keywords:** thymus, thymic cyst, mediastinal lymphoma, anterior mediastinum, mediastinal surgery

## Abstract

Thymic cysts are rare lesions (1–5% of all mediastinal masses) and, most of the times, are incidental findings. The coexistence of a lymphoma and a thymic cyst is rare. In the case reported herein, microscopic foci of a diffuse large B-cell lymphoma were identified in the wall of a resected thymic cyst. This case report adds to the current knowledge of this rare entity and highlights the necessity of early surgical resection of mediastinal cysts over watchful waiting.

## Introduction

Thymic cysts are rare lesions (1–5% of all mediastinal masses) and, most of the times, are incidental findings (1–3). The coexistence of a lymphoma and a thymic cyst is rare (4). In the case reported herein, microscopic foci of a diffuse large B-cell lymphoma were identified in the wall of a resected thymic cyst. The aim of this case is to add to the current knowledge of this rare entity and to underline the importance of surgical treatment of cystic lesions of the anterior mediastinum over watchful waiting.

## Case Report

**Figure 1:**
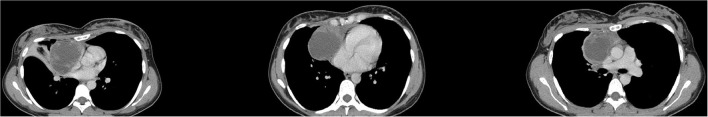
Chest computed tomography (CT) scan showing an anterior mediastinal mass with cystic features.

**Figure 2:**
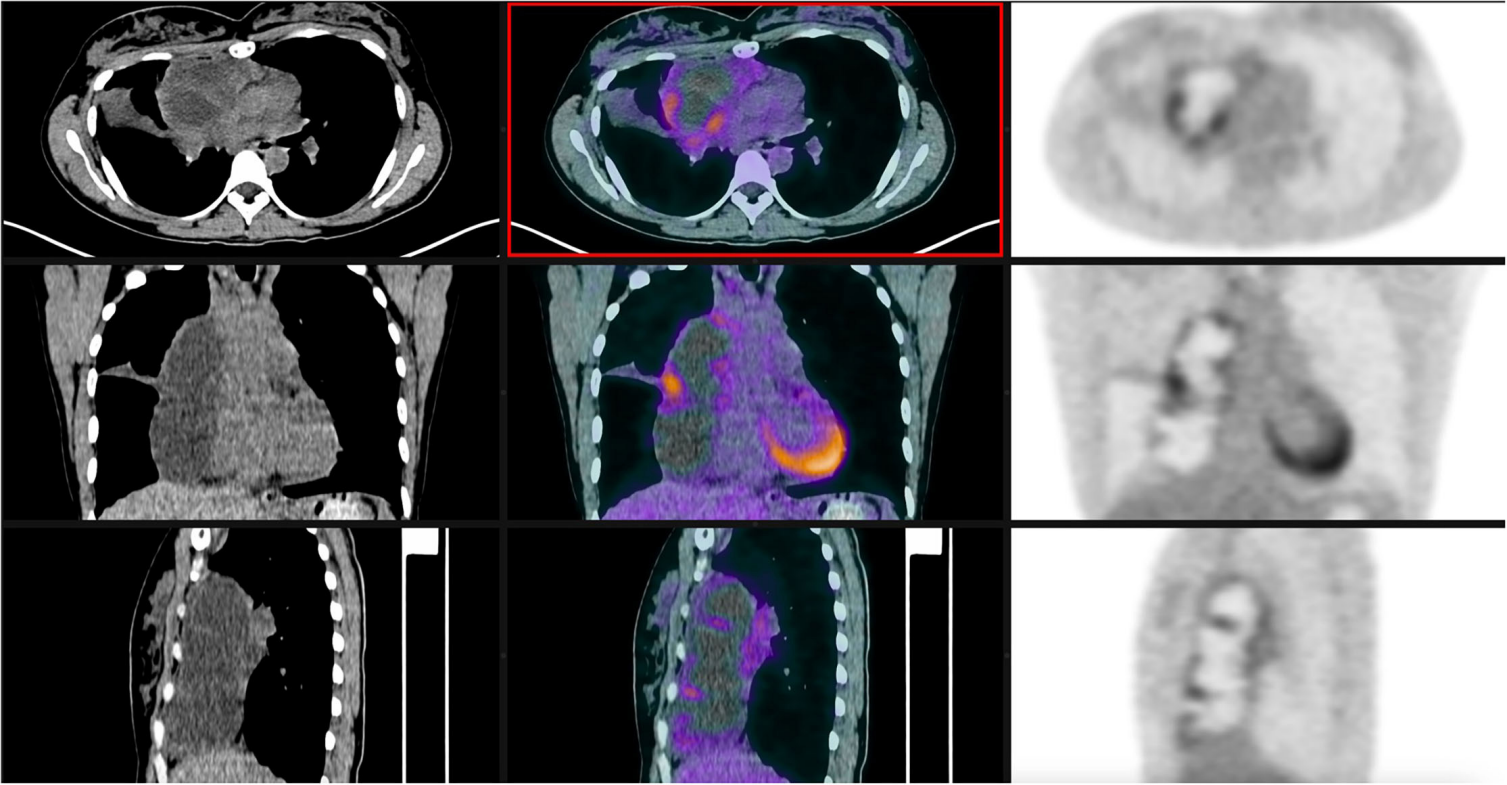
^18^FDG PET-CT scan showing hypermetabolic activity at the level of the mass.

**Figure 3:**
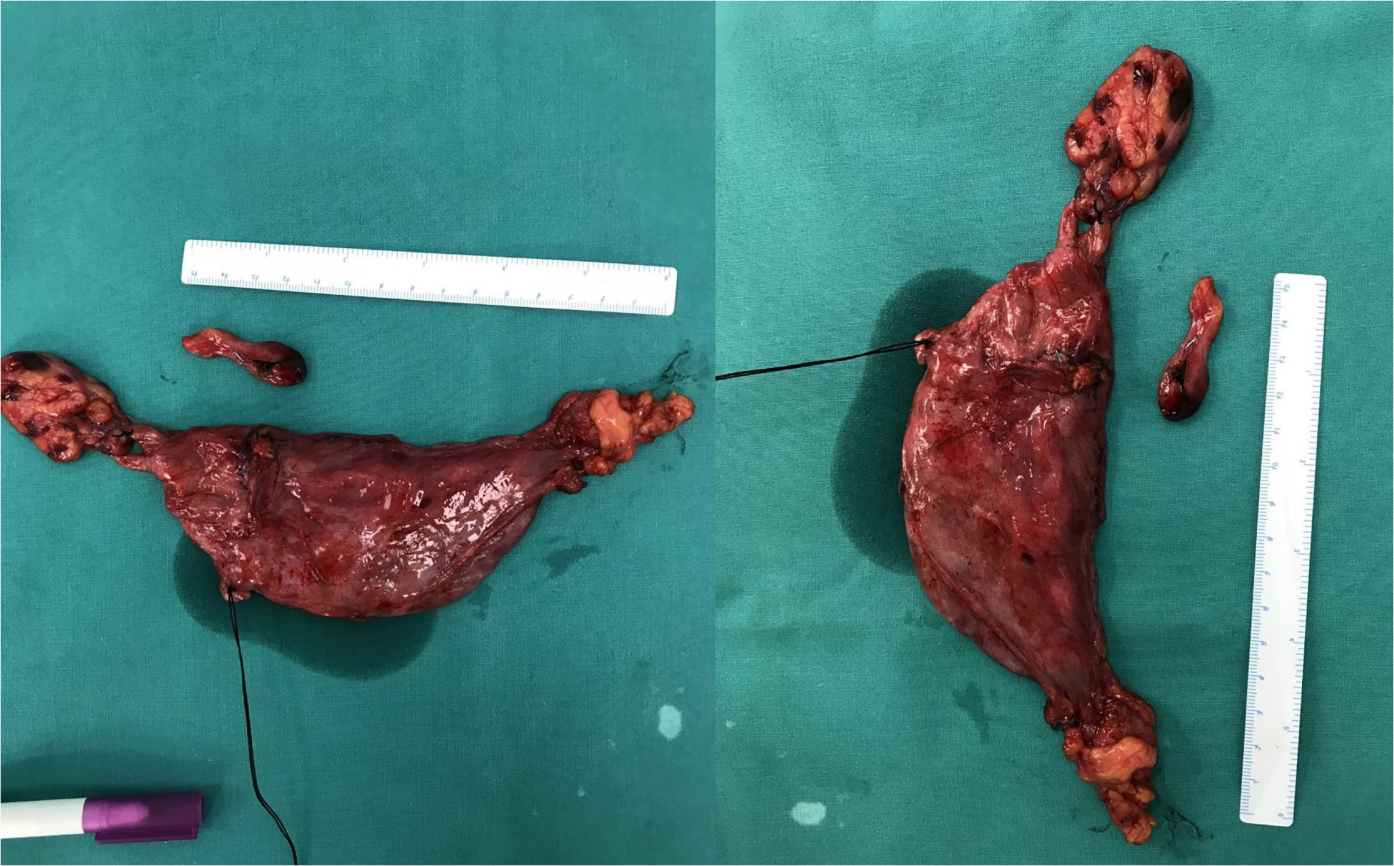
The resected specimen.

A 32-year-old female patient with unremarkable medical history had a fortuitous diagnosis of an anterior mediastinal mass. A chest CT scan showed a voluminous mass (82 mm on long axis) with cystic features situated in the anterior mediastinum. There were no signs of invasion of the adjacent structures (Figure 1). An ^18^fluorodeoxyglucose PET-CT scan showed hypermetabolic activity in the periphery of the mass (Standard uptake value max 5.1) with no other local or distant hyperfixation (Figure 2). The lesion was deemed resectable, and upfront surgery was scheduled. Because of the volume of the mass, access was gained through a full median sternotomy. A total thymectomy with resection of the mass was performed with no intraoperative complications (Figure 3). The postoperative course was uneventful, and the patient was discharged on postoperative day 4.

**Figure 4:**
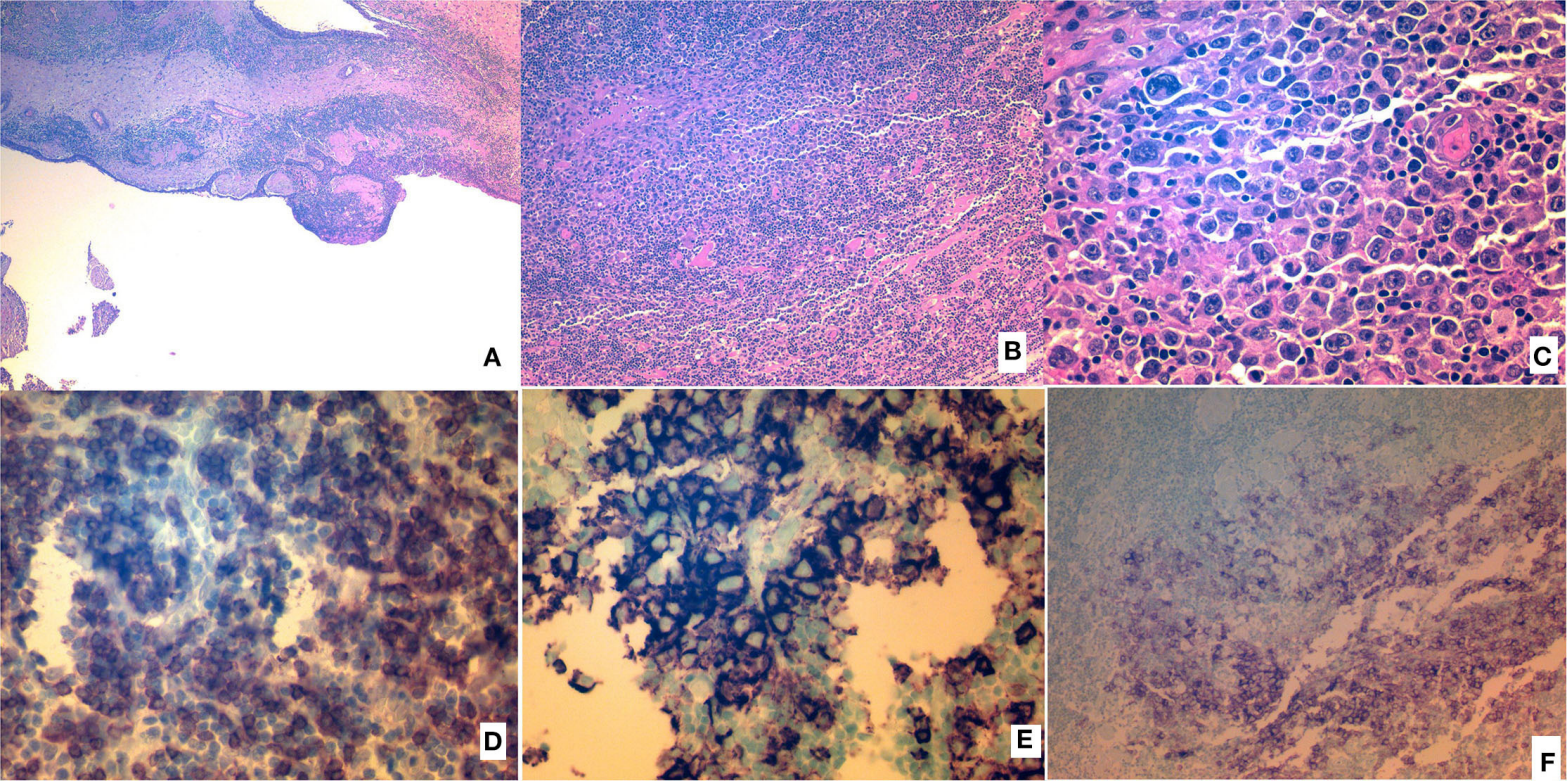
Histopathologic analysis of the resected specimen. **(A)** Hematoxylin and eosin (H&E) image of the wall and epithelium of the cyst. **(B)** Layers of neoplastic cells (H&E). **(C)** Layers of neoplastic cells (H&E), magnified. **(D)** Neoplastic cells showing CD79a positivity. **(E)** Neoplastic cells showing CD20 positivity. **(F)** Neoplastic cells showing CD30 positivity.

The histopathological analysis of the surgical specimen was compatible with a thymic cyst. However, in its wall, there were foci of neoplastic cells, which were bigger than adjacent lymphocytes. Some of the cells were even bigger; they had bigger and multiloculated nuclei that were similar to those of Hodgkin cells. Immunohistochemical analysis was negative for cytoceratin AE1/AE3, placental alkaline phosphatase, and Octamer-binding transcription factor 3/4, and positive for CD45, CD20, CD79a, and CD30. Many neoplastic cells were partially positive for p63 (Figure 4). In conclusion, the analysis was compatible with an early-stage lymphoma suggesting a primary mediastinal diffuse large B-cell lymphoma.

Taking into account the aggressive potential of the disease, the multidisciplinary tumor board decided to administrate adjuvant treatment, even though resection margins were free from tumor invasion and thymic cyst involvement by the neoplastic tissue was microscopic. Consequently, 6 cycles of an R-CHOP regimen (rituximab, cyclophosphamide, doxorubicin, vincristine, and prednisone) were administered.

## Discussion

Thymic cysts are rare lesions and can be unilocular or multilocular (1–9). Most congenital cysts are unilocular, and acquired cysts are frequently multilocular (7). Multilocular cysts are the result of a chronic inflammatory process. Majority of the lesions are asymptomatic, and they are discovered incidentally. However, in case of significant growth or complications, they can become symptomatic. Their predominant localization is the anterior mediastinum. Chest CT scan can safely establish the diagnosis (5). There is no consensus about their treatment, but surgical resection is the privileged strategy, since it can provide definitive histological diagnosis and avoid potential complications such as infection, spontaneous rupture, or malignant transformation (3).

The coexistence of a lymphoma and a thymic cyst is a rare entity, with few cases reported in the literature (7–9). The exact etiopathology is largely unknown. There are scarce reports on different types of lymphomas with the predominance of Hodgkin's disease. In 1983, Smith et al. described a case, similar to the case reported herein, in a 14-year-old boy (8). There were foci of Hodgkin's disease (nodular sclerotic morphology) in the form of nodules in the cyst wall. The authors concluded that Hodgkin's disease should be part of the differential diagnosis of any mediastinal cyst especially in younger individuals (8).

Neoplastic cells can be organized in nodules, and they are bigger than normal lymphocytes, as in the case reported by Haque et al. (4). The cyst can contain hyperplastic thymic epithelium. Calcification is the result of chronic inflammatory process. In the case reported by Tabata et al. a mediastinal gray zone lymphoma originated from CD20+CD30+ Hodgkin cell-like cells and was associated with a previously resected thymic cyst. This type of cells proliferated together with thymic epithelial cells (7).

## Conclusions

The coexistence of a thymic cyst and a lymphoma is rare and, most of the time, is discovered during histopathological examination of the resected specimen. The exact pathogenetic mechanism is unknown. This case report adds to the current knowledge of this rare entity and highlights the necessity of early surgical resection of mediastinal cysts over watchful waiting.

## Data Availability Statement

The original contributions presented in the study are included in the article/supplementary material, further inquiries can be directed to the corresponding author/s.

## Ethics Statement

Written informed consent was obtained from the patient for the publication of any potentially identifiable images or data included in this article.

## Author Contributions

AA: data analysis and manuscript writing. IP: literature research and manuscript writing. KG: synthesis of data, editing, and manuscript writing. SM: manuscript writing and final approval. All authors contributed to the article and approved the submitted version.

## Conflict of Interest

The authors declare that the research was conducted in the absence of any commercial or financial relationships that could be construed as a potential conflict of interest.

## Publisher's Note

All claims expressed in this article are solely those of the authors and do not necessarily represent those of their affiliated organizations, or those of the publisher, the editors and the reviewers. Any product that may be evaluated in this article, or claim that may be made by its manufacturer, is not guaranteed or endorsed by the publisher.
